# Lipid Nanoparticle Delivery of CAR276 mRNA Enables Antigen-Specific Cytotoxicity in T Cells Against Glioblastoma Cells

**DOI:** 10.3390/biomedicines14102365

**Published:** 2026-10-16

**Authors:** Fatemeh Zahedipour, Sogand Sadeghianfard, Silas Wisser, Meshkat Dinarvand, Denise Steiner, Anjana Ramesh, Paul Trosien, Josef Leibold, Markus Mezger, Tahereh Mohammadian Gol

**Affiliations:** 1 Department of Hematology/Oncology, University Children’s Hospital, University of Tübingen, 72076 Tübingen, Germany; 2 Cluster of Excellence iFIT (EXC2180) “Image-Guided and Functionally Instructed Tumor Therapies”, University of Tübingen, 72076 Tübingen, Germany; 3 Department of Medical Oncology and Pneumology, University Hospital Tübingen, 72076 Tübingen, Germany; 4 Institute of Pharmaceutical Technology and Biopharmaceutics, University of Münster, 48149 Münster, Germany

**Keywords:** lipid nanoparticles, CAR-T cell engineering, CD276 (B7-H3), glioblastoma, mRNA delivery

## Abstract

**Background/Objectives:** Glioblastoma multiforme (GBM) remains the most aggressive and treatment-refractory primary brain tumor, with current therapeutic modalities providing minimal survival improvement. Chimeric antigen receptor (CAR)-based immunotherapies succeeded in hematologic malignancies but are constrained in solid tumors by tumor heterogeneity, immunosuppressive microenvironment, and delivery challenges. CD276 (B7-H3), a pan-cancer antigen overexpressed in gliomas and correlated with poor patient outcomes, represents a compelling immunotherapeutic target. **Methods**: Here, we developed a non-viral lipid nanoparticle (LNP)-based platform for transient CAR mRNA delivery to T cells and evaluated its application for generating CD276 (B7-H3)-targeted CAR-T cells against GBM. An in-house-assembled T-mixer incorporating polyether ether ketone (PEEK) tubing was integrated with a dual-syringe-pump system for controlled LNP production. **Results**: LNPs formulated with clinically relevant ionizable lipids, SM-102 and DLin-MC3-DMA, demonstrated high encapsulation efficiency and effective mRNA delivery. Cell-type-dependent expression kinetics were further characterized following LNP-mediated reporter mRNA delivery in K562, NK, NK92, and T cells, revealing differences in transgene expression across cell populations. LNP-mediated delivery of CD276 CAR mRNA enabled generation of CAR-expressing T cells that exhibited antigen-specific cytotoxicity against CD276-positive LN229 GBM cells in vitro. **Conclusions**: Collectively, this study demonstrates the feasibility of an adaptable LNP-based platform for non-viral CAR mRNA engineering of T cells and CD276-targeted cytotoxicity against GBM cells in vitro. The combination of transient CAR expression and LNP-mediated mRNA delivery provides a foundation for further optimization and evaluation in preclinical in vivo models.

## 1. Introduction

Gliomas represent the most common type of primary brain malignancy [1,2]. They arise from glial cells, including astrocytes, oligodendrocytes, and ependymal cells, which provide structural and functional support to neurons. Histologically, gliomas are classified into six subtypes, among which glioblastoma multiforme (GBM), a grade IV glioma, is the most frequent and aggressive malignant primary brain tumor. Originating from astrocytes, the most abundant glial population in the central nervous system (CNS), GBM is characterized by rapid progression, therapeutic resistance and poor patient prognosis. Despite standard treatment consisting of maximal safe surgical resection followed by radiotherapy in combination with temozolomide, tumor recurrence is nearly inevitable [3,4]. This refractory nature is largely attributed to tumor heterogeneity, genomic instability, and the capacity of GBM cells to evade antitumor mechanisms, complicating the treatment of both primary and recurrent disease [5].

Various immunotherapeutic approaches have been evaluated in clinical trials for GBM, but their overall impact on survival has been modest. Recently, increasing attention has been directed toward chimeric antigen receptor (CAR)-based therapies, in which autologous T cells are engineered to recognize tumor-associated antigens. This strategy has produced remarkable results in hematologic malignancies [6], yet its success in solid tumors, including GBM, has been limited. Over the past decade, CAR-T cell therapy has emerged as a major focus in GBM research. In the United States alone, results from 10 clinical trials have been reported, highlighting both encouraging findings and significant barriers to efficacy [7].

CD276, also known as B7-H3, was initially identified as a co-stimulatory/co-inhibitory molecule [8]. As a member of the B7 immunoregulatory family, CD276 can promote the activation of CD4^+^ and CD8^+^ T cells, thereby enhancing cellular immune responses [8]. While originally described as a co-stimulatory factor in T cell-mediated allograft rejection, accumulating evidence indicates that CD276 is strongly associated with tumor progression and metastasis, with high expression observed across a wide range of cancers [9,10]. Furthermore, CD276 has been identified as a marker of micrometastatic tumor cells [11]. Its upregulation is correlated with reduced immune cell infiltration and suppression of lymphocyte activity, particularly natural killer (NK) and CD8^+^ T cells [10,12]. In gliomas, CD276 overexpression has been documented, and preclinical studies have shown that CD276-directed CAR-T cells can effectively suppress tumor growth, suggesting a potential new therapeutic strategy [13].

Although most CAR-based strategies have focused on T cells, NK cells are emerging as a promising alternative due to their strong antitumor properties and favorable safety profile in allogeneic settings, which may circumvent limitations of autologous CAR-T cell therapies [14]. To date, the majority of CAR NK studies have employed NK92 cells, an IL-2-dependent, continuously proliferating human NK cell line that exhibits many features of activated NK cells, except for FcγRIII (CD16) expression [15]. Preclinical models have demonstrated that NK92 cells exert robust and durable cytotoxicity against both hematological and solid tumors [15,16]. Moreover, clinical trials confirmed the safety of irradiated NK92 cells, with some patients achieving durable clinical responses [17]. Building on these findings, several clinical studies have explored CAR NK cell therapy for GBM using both autologous and allogeneic NK cells [18].

Lipid nanoparticles (LNPs) have recently emerged as highly effective carriers for RNA-based therapeutics, offering protection to RNA cargo while enhancing cellular uptake [19]. Clinically, LNP platforms have been validated with FDA-approved therapies such as Onpattro for hereditary transthyretin amyloidosis [20] and the LNP-based mRNA COVID-19 vaccines [21], which have revolutionized the field of RNA medicine. Compared with viral vectors, LNPs present multiple advantages in nucleic acid delivery [22,23] including the capacity for repeated dosing, the ability to encapsulate larger RNA molecules, broad intratumoral distribution, and transient expression of mRNA, features that reduce nuclease degradation and minimize off-target toxicities [24]. Furthermore, LNPs with optimized physicochemical properties can enhance blood–brain barrier penetration and target tumor-specific ligands, improving therapeutic precision while reducing systemic toxicity compared with conventional chemotherapeutics [25,26,27]. Recently, Capstan Therapeutics has launched Phase 1 trials for CPTX2309, a pioneering CD8-targeted LNP that delivers anti-CD19 CAR mRNA directly to T cells (NCT06917742). This represents the first in vivo CAR-T cell therapy product to reach the clinic and marks a significant milestone in non-viral, cell-targeted gene delivery.

Despite extensive investigation of CD276 directed CAR-T cells, an efficient non-viral platform for transient generation of CD276 CAR-T cells using LNP-mediated CAR mRNA delivery has, to our knowledge, not yet been reported in GBM. In this context, we developed LNP formulations using clinically validated ionizable cationic lipids for efficient and safe delivery of CD276 CAR mRNA into T cells. These engineered cells demonstrated potent activity against LN229 GBM cell lines in vitro, providing a strong foundation for the future in vivo evaluation of this strategy.

## 2. Materials and Methods

### 2.1. Lipid Nanoparticle Production

Lipid nanoparticles (LNPs) were prepared using either SM-102 or DLin-MC3-DMA as the ionizable lipid. The lipid components were combined at a molar ratio of 50:10:38.5:1.5 (ionizable lipid:DSPC:cholesterol:DMGPEG2000), with a total lipid concentration of 6 mM. SM-102 and DLin-MC3-DMA was obtained from Broadpharm, San Diego, CA, USA. DSPC, cholesterol and DMG-PEG2000 was obtained from Merck, Darmstadt, Germany. A thin lipid film was created by evaporating the solvents with a rotary evaporator at 37 °C (IKA-Werke GmbH & Co. KG, Staufen im Breisgau, Germany). For production of the LNPs, lipid films were dissolved in absolute ethanol, and the mRNA was dissolved in sodium citrate buffer (50 mM, pH 4) to a total volume of 900 µL and the proper concentration to reach an N/P ratio of 6. LNP formation was performed using a self-engineered PEEK T-mixer coupled to two syringe pumps (Longer, Baoding, China). The lipid-containing ethanol phase and RNA-containing aqueous phase were delivered separately into the T-mixer at flow rates of 8 mL/min and 24 mL/min, respectively. To reduce the ethanol concentration and stabilize the particles, a post-dilution strategy was carried out by directly adding sodium citrate buffer. The LNPs were concentrated by centrifugal filtration to reach the final volume of 900 µL (final formulation volume). The formulation buffer was exchanged to 20 mM Tris buffer containing 10% sucrose (pH 7.4) by dialysis. Finally, LNPs were filtered through a 0.2 µM syringe filter and stored at −20 °C or −80 °C.

The T-mixer was assembled in house using commercially available components made of polyether ether ketone (PEEK). The T-mixer consisted of a T-connector (KNAUER, Berlin, Germany) with a 1/16 inch outer diameter (OD), Luer adapters (10-32, ETFE; female Luer to male 10-32, Sigma-Aldrich, Inc., St. Louis, MO, USA), and couplings (KNAUER, Berlin, Germany) for connecting two capillaries with a 1/16 inch OD. PEEK tubing (Sigma-Aldrich, Inc., St. Louis, MO, USA) was used for delivery of the aqueous and lipid phases. The tubing used for the lipid phase had dimensions of 1/16 inch OD × 0.010 inch inner diameter (ID), whereas the tubing used for the aqueous phase had dimensions of 1/16 inch OD × 0.030 inch ID.

### 2.2. Lipid Nanoparticle Characterization

Encapsulation efficiency (EE) was measured using Promega QuantiFluor RNA system. The LNPs were diluted 1:1 in TE buffer. Diluted LNP samples were mixed 1:1 with TE buffer or 2× Triton and incubated for 10 min at 37 °C. EE [%] was determined by:

(1) $$\mathrm{EE}\left[\%\right]=1-\frac{\mathrm{Unencapsulated RNA}(\mathrm{LNP}-\mathrm{TE mix})}{\mathrm{total RNA}(\mathrm{LNP}-\mathrm{Triton mix})}\times 100$$

Dynamic light scattering (DLS) analysis was conducted using a Zetasizer Nano instrument (Malvern Instruments, Malvern, UK) to determine the hydrodynamic diameter (z-average), polydispersity index (PDI), and zeta potential (surface charge) of the lipid nanoparticles. Measurements were performed in sample triplicates. Finally, transmission electron microscopy (TEM) (Zeiss, Jena, Germany) was performed using negative staining to determine the morphological characteristics of LNPs.

### 2.3. In Vitro-Transcribed mRNA Production

The pCS2-DsRed and pVAX-CD276 CAR plasmids were used as templates for in vitro transcription (IVT) of DsRed and CD276 CAR mRNAs, respectively. The plasmids were linearized using the appropriate restriction enzymes downstream of the transcriptional template and purified prior to IVT. The linearized DNA templates were used for mRNA synthesis with the HiScribe SP6 High Yield RNA Synthesis Kit and HiScribe T7 High Yield RNA Synthesis Kit (New England Biolabs, Ipswich, MA, USA) for DsRed and CD276 CAR mRNA production, respectively. IVT reactions were performed according to the manufacturer’s instructions. A schematic representation of the DsRed and CD276 CAR mRNA constructs and their respective structural elements is shown in Supplementary Figure S1. Following transcription, template DNA was removed by DNase treatment, and the resulting mRNA was purified using a lithium chloride (LiCl)-based precipitation method. The concentration and purity of the purified mRNA were determined using a NanoDrop spectrophotometer, and RNA integrity was assessed by agarose gel electrophoresis. Purified mRNAs were aliquoted and stored at −80 °C until further use. All procedures were performed under RNase-free conditions [28].

### 2.4. Cell Culture

All cells were cultured at 37 °C with 5% CO_2_ atmosphere. K562 cells were purchased from Sigma-Aldrich and were cultured in RPMI 1640 supplemented with 10% FBS, 1% L-glutamine and 1% penicillin/streptomycin. LN229 cell lines were obtained as a gift from Dr. Josef Leibold, Department of Medical Oncology and Pneumology, University Hospital Tübingen, Tübingen, Germany and were cultured in DMEM supplemented with 10% FBS and 1% penicillin/streptomycin. Heparinized peripheral blood samples were obtained from healthy donors after informed consent. NK cells were isolated and expanded according to previously published protocol [29]. T cells were purified using the Pan T Cell Isolation Kit (Miltenyi Biotec, Bergisch Gladbach, Germany) and stimulated with CD3/CD28 T cell Activator Dynabeads (Invitrogen, Carlsbad, CA, USA) as previously described [30], then cultured in TexMACS medium (Miltenyi Biotec, Bergisch Gladbach, Germany) supplemented with 1% penicillin/streptomycin, 10 ng/mL Interleukin 7, and 5 ng/mL Interleukin 15. NK92 cells were purchased from the American Type Culture Collection (ATCC, Manassas, VA, USA) and were cultured in MEMa GluMax supplemented with 20% FBS, 1% penicillin/streptomycin, and 100 Unit/mL Interleukin 2.

### 2.5. Transfection and Flow Cytometry

LNP transfection was performed by adding the respective LNP formulations directly to the cells. Cells were thawed 2 to 3 days prior to transfection and seeded at a concentration of 1 × 10^6^ cells in 1 mL of appropriate culture medium. LNPs were added directly to the cells at defined doses of 0.5, 2.5, and 5 µg, corresponding to final concentrations of 0.5, 2.5, and 5 µg/mL, respectively. For T cells, Apolipoprotein E4 was supplemented at a concentration of 1.5 µg/mL in the culture medium. Electroporation was performed using the ExPERT GTx™ electroporation system (MaxCyte, Rockville, MD, USA) with R-50x3 cuvettes. Briefly, 5 µg of mRNA was electroporated into 2.5 × 10^6^ cells. The K562 and Program 3 electroporation programs were used for K562 cells and T cells, respectively.

The expression of mRNAs and cell surface markers were characterized using fluorophore-labeled antibodies including anti-CD276-PE, CD56-APC, and CD34-PE from Miltenyi Biotec, Bergisch Gladbach, Germany and CD3-FITC from Biolegend, San Diego, CA, USA. An isotype control was used for each fluorophore used. Samples were run on a BD FACSCalibur™ flow cytometer (BD Biosciences, San Diego, CA, USA) and analyzed with the FlowJo version 10.10 software (FlowJo LLC, Ashland, OR, USA).

### 2.6. Cytotoxicity Assay

The cytotoxic activity of effector cells against target cells was assessed using a luciferase-based cytotoxicity assay. Both LN229 cell lines express luciferase and can metabolize luciferin, which was added into the assay. Briefly, 1 × 10^4^ target cells were suspended in 100 µL of assay medium (RPMI supplemented with 1% L-glutamine and 1% penicillin/streptomycin). Effector cells (in indicated ratios) were resuspended in 50 µL of the same medium and co-cultured with target cells in white 96-well plates. Cytotoxicity was quantified 16 h after co-culture by measuring luminescence using a TECAN Spark reader immediately after luciferin addition. To calculate the specific lysis by effector cells, the following formula was used:

(2) $$\%\;\mathrm{specific lysis}=\frac{\mathrm{Measured value}-\mathrm{Spontaneous lysis mean}}{\mathrm{Maximum killing}-\mathrm{Spontaneous lysis mean}}\times 100$$

The measured value represents the signal obtained from target cells incubated with effector cells under the experimental conditions. Spontaneous lysis mean represents the mean signal obtained from target cells cultured in the absence of effector cells. Maximum lysis mean represents the mean signal obtained from target cells treated with 1% Triton X-100 to induce maximal target-cell lysis.

### 2.7. Statistical Analysis

All experiments were performed using at least three independent experiments for cell lines and three independent donors for primary cells. Statistical analyses were conducted using Prism 8.0 (GraphPad Software, La Jolla, CA, USA) on datasets obtained from a minimum of three biological and experimental replicates. The normality of the data was assessed using the Shapiro–Wilk test. Flow cytometry results were processed and analyzed with FlowJo version 10.10 (FlowJo LLC, Ashland, OR, USA).

## 3. Results

### 3.1. Preparation and Physicochemical Characterization of LNPs

**Figure 1:**
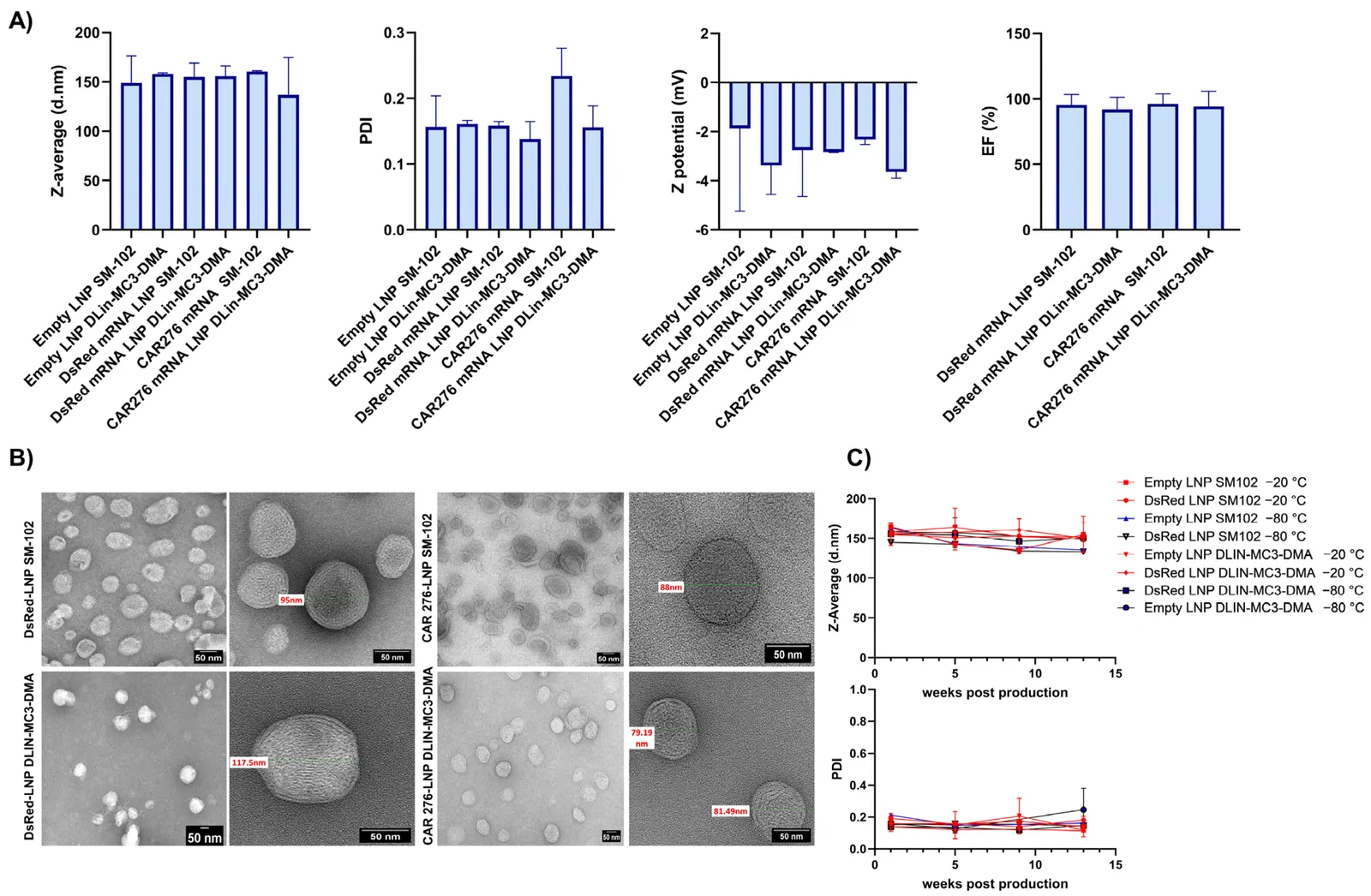
Physicochemical characteristics of LNPs. (**A**) The size (z-average), polydispersity index (PDI), and surface charge were measured for at least two independent LNP productions, with three measurements performed for each production. Encapsulation efficiency (EE) was determined for four independent LNP productions. (**B**) Representative TEM image of LNPs formulated with two different ionizable lipids for the encapsulation of DsRed and CAR276 mRNAs. (**C**) Effect of storage conditions on LNP particle size. Lipid nanoparticles (LNPs) were stored at either −20 °C or −80 °C following production, and particle size was monitored over time to evaluate size stability under different storage conditions. Measurements were performed at defined time points after production. Data represent the mean particle size measured by dynamic light scattering (DLS) from two independent LNP productions, with three measurements performed for each production.

In this study, two ionizable lipids, SM-102 (a component of the FDA approved Moderna mRNA-1273 vaccine) and DLin-MC3-DMA (a component of the FDA-approved siRNA therapeutic Onpattro^®^) were selected for the formulation of lipid nanoparticles. In brief, the ethanolic phase (lipids) and aqueous phase (DsRed mRNA and CAR276 mRNA with 678 and 2569 nucleotides length, respectively) were mixed using a T-mixer connected to two syringe pumps to produce mRNA-LNPs. In the following step, the mRNA-LNPs were unconcentrated using Amicon ultrafiltration and dialyzed against a 20 mM Tris-sucrose (10%) to remove ethanol. The synthesized LNPs were subsequently analyzed to determine their size, PDI, and surface charge by dynamic light scattering (DLS). The measured diameters, expressed as the z-average, varied between 110 and 166 nm, with PDIs remaining under 0.2 (Figure 1A), indicating that the formulations are monodispersed. Moreover, the result of the encapsulation efficiency measurement showed over 90% encapsulation efficiency for DsRed and CAR mRNA-LNPs (Figure 1A). Transmission electron microscopy (TEM) images revealed the characteristic morphology and nanoscale structure of the lipid nanoparticles (Figure 1B).

In addition, a stability study was performed to check the effect of storage conditions on the size of the LNPs. In this study, LNPs were stored at either −20 °C or −80 °C, and their particle size was monitored over time post-production to evaluate stability and quality. No significant changes in size were detected for any formulation across both storage conditions over a period of approximately three months (Figure 1C).

**Figure 2:**
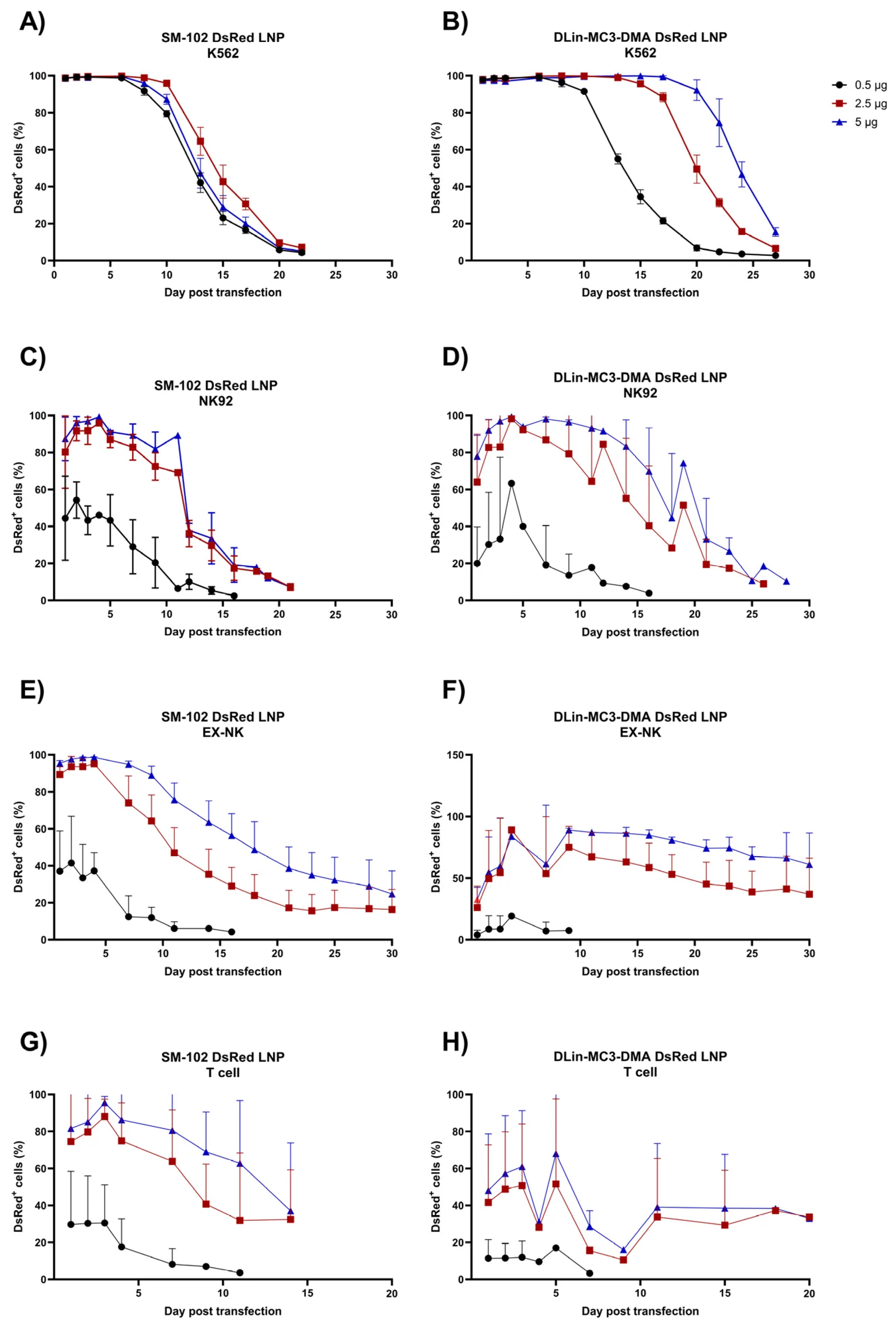
Cell type-specific expression of DsRed mRNA-LNPs. Various cell types were transfected with DsRed mRNA-LNPs formulated using either SM-102 or DLin-MC3-DMA as the ionizable lipid. Panels show: (**A**,**B**) K562 cells (*n* = 3, independent experiments), (**C**,**D**) NK92 cells (*n* = 3, independent experiments), (**E**,**F**) expanded NK cells (*n* = 3, independent donors), and (**G**,**H**) primary T cells (*n* = 4 for SM-102, *n* = 3 for DLin-MC3-DMA, independent donors). DsRed expression was assessed by flow cytometry, and results are shown as the percentage of DsRed-positive cells. Data are presented as mean ± standard deviation (SD) from independent experiments. Non-transfected cells were used as control.

To evaluate the functionality of the synthesized lipid nanoparticles, different cell types comprising human K562 erythroleukemia cells, the NK92 natural killer cell line, as well as ex vivo expanded NK cells and primary T cells from healthy donors were transfected with lipid nanoparticles containing DsRed mRNA. The expression of the DsRed protein was monitored by flow cytometry over subsequent days until the proportion of DsRed-positive cells declined below 20% (Figure 2). Cells were transfected using three distinct mRNA concentrations (0.5, 2.5, and 5 µg). Untransfected cells were used as controls. Transfection with both DsRed-loaded LNP formulations resulted in greater than 99% DsRed-positive cells within the first three days across all tested mRNA concentrations in K562 cells (Figure 2A,B). However, DsRed LNPs formulated with DLin-MC3-DMA exhibited higher cellular toxicity compared with those formulated with SM-102 (Supplementary Figure S2). In the low-dose (0.5 µg) transfected cells, the mRNA expression level started to decrease earlier, with no cytotoxic effect on cells. In NK92 cells, as well as ex vivo expanded NK and primary T cells, a difference in the proportion of positive cells was observed between the low and high doses. Donor-to-donor variability in the proportion of positive T cells was observed at higher doses of DsRed-DLin-MC3-DMA LNPs (Figure 2C–H).

In addition, LNPs encapsulating DsRed mRNA were stored at −20 °C and subsequently used to transfect K562 cells 11 weeks post-production. DsRed expression was quantified by flow cytometry and compared to transfection results obtained one week post-production (Supplementary Figure S3). The DsRed SM-102 LNPs demonstrated stable DsRed expression levels over time, with no significant difference observed between week 1 and week 11 post-production. In contrast, DsRed DLIN-MC3-DMA LNPs exhibited a modest reduction in DsRed expression over time, with a decline to below 10% DsRed-positive cells occurring 3 to 7 days earlier than observed in the week 1 post-production transfections (Supplementary Figure S3).

### 3.2. Cell Type-Specific Expression of CAR mRNA Loaded LNPs

**Figure 3:**
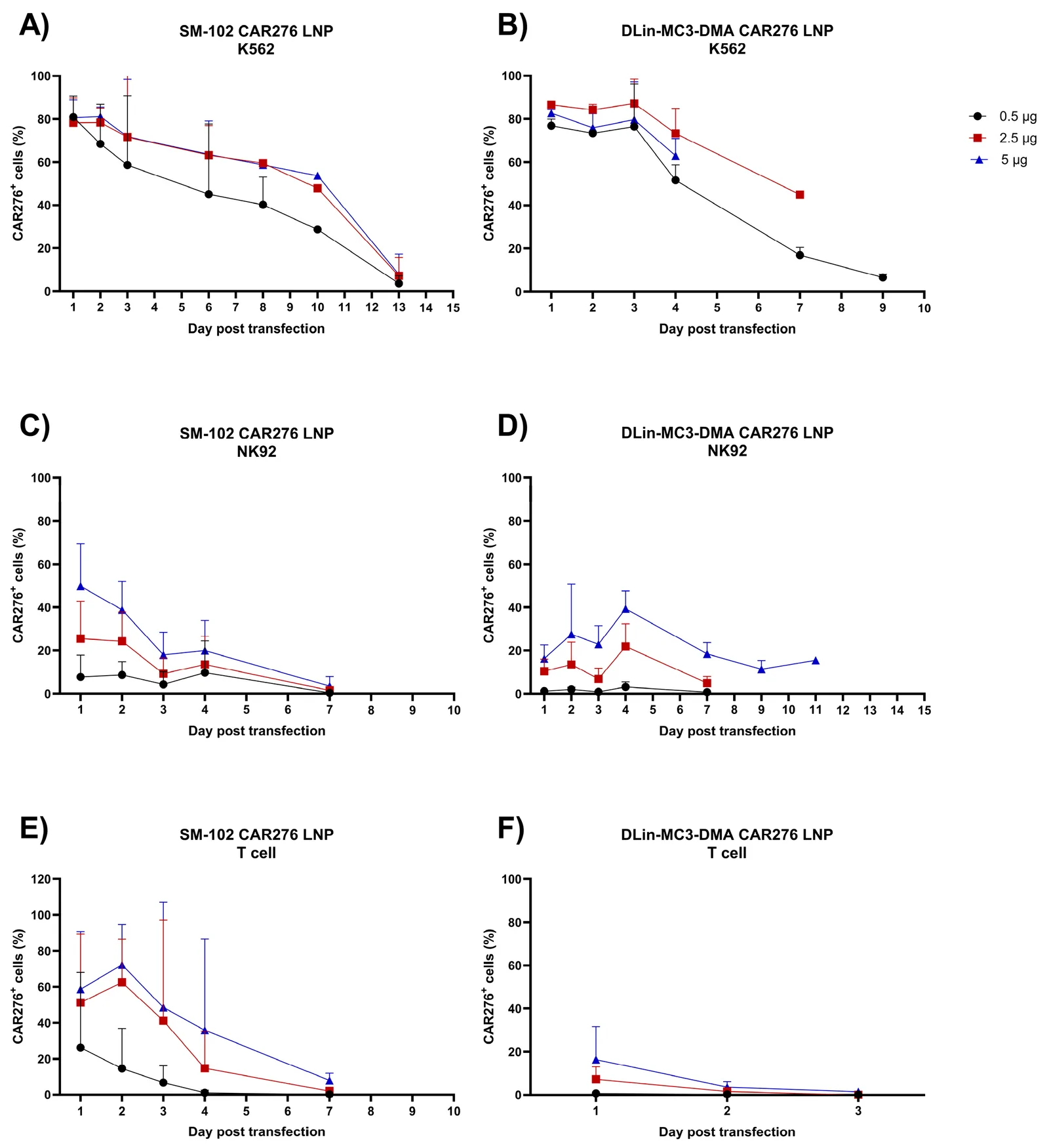
Cell type-specific expression of CAR276 mRNA-LNP. Various cell types were transfected with CAR276 mRNA-LNPs formulated using either SM-102 or DLin-MC3-DMA as the ionizable lipid. Panels show: (**A**,**B**) K562 cells (*n* = 3, independent experiments), (**C**,**D**) NK92 cells (*n*=3, independent experiments) for and (**E**,**F**) T cells (*n* = 3, independent donors). CAR276 expression was assessed by flow cytometry. Untransfected cells were used as the experimental control, and an isotype control was included for flow cytometry analysis. Results are expressed as the percentage of CAR276-positive cells and presented as the mean ± standard deviation (SD).

To assess whether the kinetics observed with DsRed mRNA were maintained with our functional therapeutic transgene (CD276 CAR mRNA), we transfected the same four cell types with LNP-formulated, mRNA-encoding CAR constructs. Across all cell types, CAR mRNA expression was lower and less stable than the DsRed control (Figure 3). In K562 cells, CAR expression was high during the first 1–3 days post-transfection but declined rapidly to <5% of cells by day 13 in SM-102-based LNPs. In DLin-MC3-DMA-based LNP-transfected cells treated with a high dose of LNPs, cell viability declined after 4 days (Figure 3A,B).

In NK92 cells, CAR expression levels were lower than in K562 cells from the outset and decreased more quickly (Figure 3C,D). In ex vivo expanded NK cells, no detectable CAR expression was observed with any of the tested LNP formulations. In contrast, primary T cells showed CAR expression, with higher levels when formulated in SM-based LNPs compared to DLin-MC3-DMA-based LNPs (Figure 3E,F). These results suggest that the CAR mRNA expression might be less stable than DsRed in most cell types, with pronounced formulation and cell type-specific effects on expression efficiency. Additionally, donor-to-donor variability was observed in CAR expression across the three LNP-CAR concentrations formulated with SM-102. At day 1, CAR expression ranged from 0.8 to 74%, 22 to 94%, and 32 to 94% for 0.5, 2.5, and 5 µg LNP-CAR, respectively (Figure 3E).

### 3.3. Antigen-Specific Cytotoxicity of T Cells Transfected with CD276 CAR mRNA LNP Against Glioblastoma Cells in Vitro

**Figure 4:**
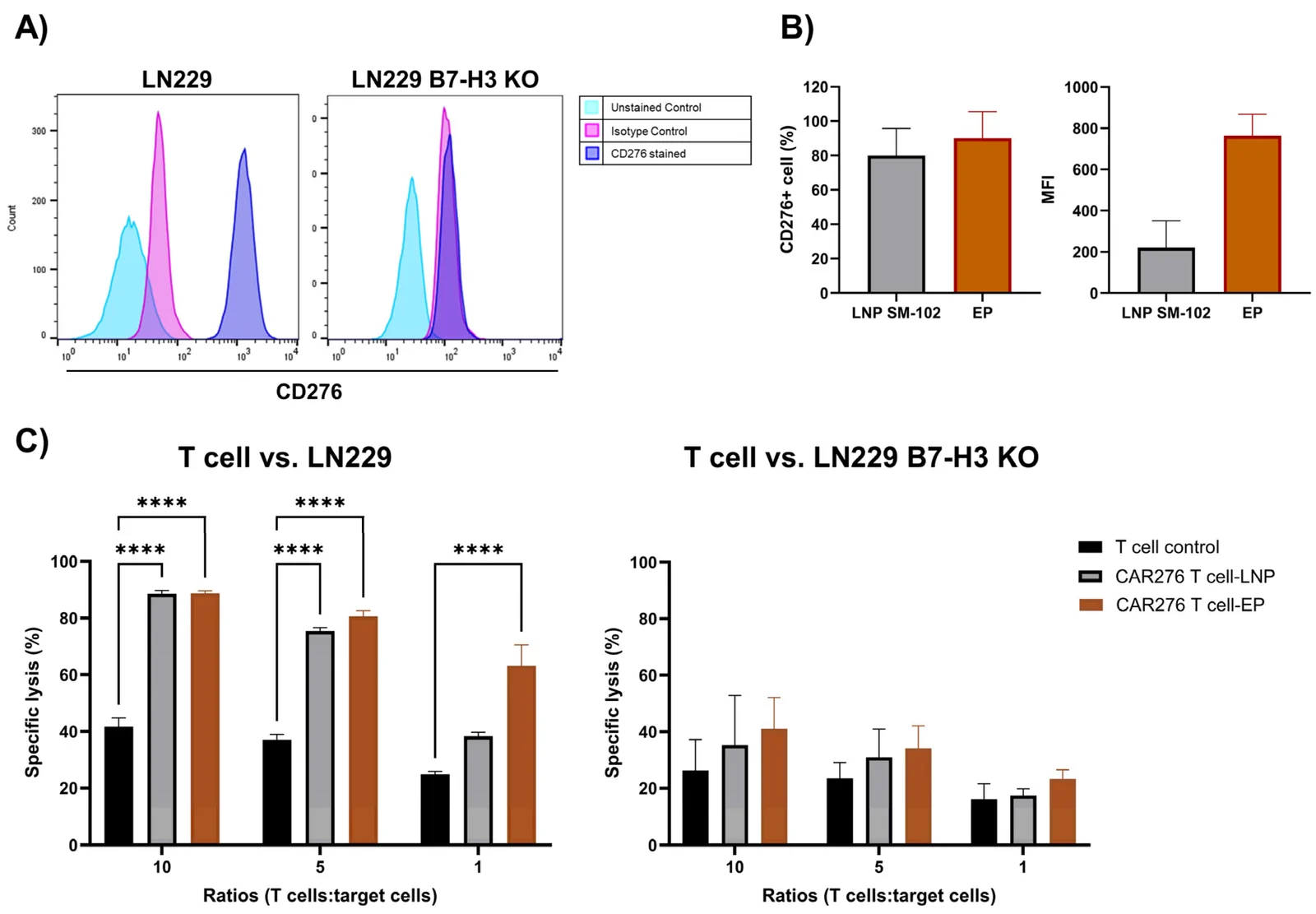
Antigen-specific cytotoxicity of CAR276-expressing T cells against glioblastoma cells. (**A**) Surface expression of the CD276 (B7-H3) antigen on LN229 glioblastoma cells and LN229 B7-H3 knockout cells, measured by flow cytometry. (**B**) Number of CD276^+^ cells and mean fluorescence intensity (MFI) in T cells from three independent donors. (**C**) Human T cells were transfected with CAR276 mRNA using either LNPs or electroporation and subsequently co-cultured with target glioblastoma cells in vitro. Non-transfected cells were used as control. Cytotoxic activity was assessed 16 h after co-culture using a luciferase-based cytotoxicity assay. Data represent the mean ± standard deviation of three independent biological replicates (three donors), each with three technical replicates. Statistical analysis was performed using a mixed-effects model with Tukey’s multiple-comparisons test. *p* < 0.0001 (****).

To evaluate the cytotoxic efficacy of T cells following CAR-LNP transfection against glioblastoma cells, a luciferase-based cytotoxicity assay was performed. Transfected T cells, at varying effector-to-target ratios, were co-cultured with the LN229-Luc/GFP cell line (a human glioblastoma-derived cell line genetically modified to stably express firefly luciferase and green fluorescent protein). Based on the previous result demonstrating high transfection efficiency and low toxicity, the formulation using SM-102 LNPs was selected to perform the killing assay. First, the high level of CD276 antigen in these cancer cells was confirmed by flow cytometry (Figure 4A). CD276-knockout LN229 cells were included as a specificity control to assess antigen-dependent cytotoxicity. Additionally, CAR mRNA was delivered to T cells via electroporation as a comparative transfection method. Both LNP-transfected and electroporated T cells showed high CAR expression (Figure 4B). The cytotoxicity of CAR-expressing T cells was assessed 16 h after co-culture with target cells, using effector-to-target ratios of 10:1, 5:1, and 1:1. Untreated cells were used as a control for both LNP-transfected and electroporated cells. As shown in Figure 4C, CAR-expressing T cells generated by both LNP-mediated transfection and electroporation demonstrated significantly enhanced cytotoxicity against LN229 cells at all tested ratios, with the most pronounced effects observed at the 10:1 and 5:1 ratio for LNP-transfected T cells. Importantly, no antigen-specific killing was detected in co-cultures with CD276-KO LN229 cells, supporting the CD276-dependent nature of the observed cytotoxicity. These results provide direct experimental evidence that the cytotoxic activity of the generated CAR-T cells against CD276-expressing LN229 cells is dependent on CD276 expression in the target cells, as the enhanced cytotoxicity observed against parental LN229 cells was not detected when CD276-KO LN229 cells were used as target cells.

## 4. Discussion

B7 homolog 3 (B7-H3, or CD276) is a widely expressed pan-cancer antigen found across numerous tumor types. Its broad presence in various malignancies highlights its potential as a universal biomarker and therapeutic target in immuno-oncology [31,32]. Studies have reported a significant negative correlation between elevated tumor mRNA levels of B7-H3 and glioblastoma patient survival [33,34]. Moreover, it has been shown that targeting CD276 can enhance NK cell-mediated anti-cancer cytotoxicity across different cancer types. Grote et al. demonstrated that lentiviral-transduced CD276-CAR NK92 cells specifically lysed melanoma cell lines and overcame various immunosuppressive effects of the tumor microenvironment [35]. In addition, they showed that CD276-CAR-engineered NK92 cells were capable of specific and long-term elimination of neuroblastoma cells in vitro [15]. Furthermore, CD276-CAR NK92 cells exhibited targeted cytotoxicity and a significant boost in antileukemic activity compared to parental NK92 cells against acute myeloid leukemia cell lines [36]. In another study, Stefańczyk et al. showed that an Fc-optimized anti-CD276 monoclonal antibody binds to CD276 on AML cell lines and primary AML cells, inducing pronounced NK cell activation and degranulation, as evidenced by increased expression of CD69, CD25, and CD107a [37].

Our preliminary results demonstrated that lentiviral-transduced CAR NK92 cells can effectively kill glioblastoma cells in vitro (Supplementary Figure S4). It is therefore worthwhile to optimize the formulation for encapsulating this CAR mRNA in LNPs in future studies. The transient expression profile of mRNA aligns well with the short in vivo lifespan of NK cells, potentially enabling a controlled and efficient therapeutic approach. Furthermore, NK cell-based therapies can circumvent several limitations associated with CAR-T cell therapy, such as the risk of graft-versus-host disease and cytokine release syndrome, while maintaining potent antitumor activity [38].

Currently, all commercially approved CAR-T cell therapies utilize lentiviral transduction technology for their development. This approach delivers the CAR gene into T cells through lentiviral vectors, which integrate into the host cell genome, ensuring stable expression of the CAR protein and long-lasting tumor control [39,40].

One promising alternative to viral-based methods is the use of CAR mRNA delivered via lipid nanoparticles. This approach allows transient expression of CARs in T and NK cells without integrating genetic material into the genome, significantly reducing the risk of insertional mutagenesis. The use of mRNA-LNPs also enables rapid and scalable manufacturing, with the flexibility to modify CAR constructs quickly. However, because CAR expression is temporary, repeated dosing may be required to maintain therapeutic effects [41].

Importantly, in this study, CAR276 mRNA delivered via LNPs produced functional antigen-specific cytotoxicity in T cells against glioblastoma LN229 target cells, with potency comparable to electroporation-based delivery. This finding is in line with the result of Meeus et al., where they showed that B7-H3 Nanobody-based CAR-T cells were efficient in killing B7-H3-positive glioblastoma cells in vitro [31].

Moreover, we evaluated the LNP-mediated delivery of both reporter and CAR mRNA into diverse hematopoietic cell types including K562, NK92 cells, expanded primary NK cells, and primary T cells from healthy donors using two LNP formulations. Our results revealed differences in mRNA expression dynamics between cell types and two formulations. In terms of storage stability, DsRed SM-102 LNPs maintained consistent expression levels across the tested period, showing no significant difference between week 1 and week 11 post-production. By contrast, DsRed DLIN-MC3-DMA LNPs showed a gradual decrease in expression over the storage time despite no observable change in particle size (Supplementary Figure S3). Our findings are in line with other reports showing that LNPs containing DLin-MC3-DMA kept their particle size during long-term storage, while a significant drop in the mRNA encapsulation efficiency was observed, which resulted in a reduced mRNA delivery efficiency in vitro and in vivo [42,43].

The chemical structures of the ionizable lipids may help explain differences in mRNA expression stability observed between formulations. DLin-MC3-DMA contains two ester linkages, which increase its susceptibility to hydrolysis, particularly under aqueous or acidic/basic conditions. By contrast, SM-102 includes only one ester group, located between the lipid tail and the amine headgroup, while its second tail is a branched alkyl chain lacking an ester bond. This structural arrangement confers greater hydrolytic stability to SM-102 compared with DLin-MC3-DMA. Such differences in chemical stability could contribute to the reduced persistence of mRNA expression observed for DLin-MC3-DMA LNPs relative to SM-102 LNPs [44].

In terms of toxicity, DLin-MC3-DMA has been associated with slower degradation of its alkyl dioleate tail, which may promote accumulation upon repeated dosing and contribute to toxicity, as previously reported in studies of siRNA delivery [44]. By contrast, the structural features of SM-102 introduce several advantages over DLin-MC3-DMA. First, their hydrophobic chains lack unsaturated C=C bonds, reducing susceptibility to oxidative degradation and enhancing nanoparticle stability. Second, the incorporation of ester linkages within the hydrophobic chains not only strengthens interactions with endosomal membranes but also facilitates membrane fusion and endosomal escape, while simultaneously allowing enzymatic degradation by esterases, thereby improving clearance, lowering toxicity, and increasing tolerability. Finally, the higher degree of branching in their hydrophobic chains improves lipid ionization under acidic endosomal conditions, which further enhances endosomal escape efficiency and supports greater levels of mRNA protein expression [44].

The rapid decline in CAR mRNA expression compared to reporter mRNA may reflect intrinsic differences in mRNA stability, codon usage, or post-transcriptional regulation compared to the DsRed mRNA. The absence of detectable expression of CAR-LNP in expanded NK cells despite clear uptake of DiD-labeled LNPs (Supplementary Figure S5), and the preferential expression in T cells with SM-102-based LNPs, underscores the importance of both cargo sequence and delivery vehicle in achieving robust transgene expression for each cell type.

Although the CD276 dependency of cytotoxicity following LNP-mediated CAR mRNA delivery was demonstrated using parental and CD276-KO LN229 cells in this study, validation across additional GBM cell lines with different levels of endogenous CD276 expression would further strengthen the generalizability of these findings and represents an important direction for future studies. A limitation of this study is the reliance on 2D cytotoxicity assays, which do not fully recapitulate the structural and immunosuppressive features of the glioblastoma microenvironment. Future studies incorporating 3D tumor spheroid models and in vivo systems will be important to more accurately evaluate T cell infiltration, persistence, and antitumor activity. Beyond ex vivo engineering, the principles demonstrated in this study could be extended towards targeted in vivo modification of T cells. Recent advances in ligand- or antibody-decorated LNPs have shown the feasibility of directing mRNA payloads to specific immune cell subsets in vivo, enabling transient CAR expression without the need for cell isolation and expansion [45]. Such strategies could reduce manufacturing complexity, shorten production timelines, and potentially broaden patient access to CAR-T-based therapies. Incorporating cell type-specific targeting ligands, such as antibodies, into our LNP formulations could enhance delivery to hard-to-target cells while reducing off-target distribution and systemic toxicity.

## 5. Conclusions

In this study, we developed a self-engineered, syringe-pump-driven PEEK T-mixer platform for the reproducible production of LNPs for CAR mRNA delivery and generated CD276-targeted CAR-T cells that exhibited antigen-specific cytotoxicity against GBM LN229 cells. By integrating non-viral, mRNA-based CAR engineering with CD276 targeting, our approach demonstrates the feasibility of transient CAR expression. Unlike permanently engineered CAR immune cells, mRNA-mediated CAR expression is inherently temporary, potentially allowing for greater control over the duration of CAR activity while limiting prolonged exposure to CAR-mediated effects. This transient expression profile may be particularly relevant in solid tumors, where sustained CAR activation could contribute to toxicity or T cell dysfunction. However, the optimal duration and level of CAR expression required to achieve sustained antitumor activity in GBM remain to be established. The present study demonstrates the feasibility of LNP-mediated CAR mRNA delivery and CD276-dependent cytotoxicity in vitro; however, the therapeutic potential of this approach remains to be established. In vivo studies will be necessary to determine therapeutic efficacy, safety, and translational applicability. Collectively, these findings support further investigation of transient mRNA-based CAR engineering as a potential approach for future immunotherapeutic strategies against GBM.

## Data Availability

The original contributions presented in this study are included in the article/Supplementary Materials. Further inquiries can be directed to the corresponding author.

## Supplementary Materials

The following supporting information can be downloaded at: https://www.mdpi.com/article/10.3390/biomedicines14102365/s1, Figure S1: Schematic representation of the CD276-CAR and DsRed mRNA constructs; Figure S2: Toxicity of LNP formulations in different cell types; Figure S3: Effect of storage on DsRed mRNA-LNP expression in K562 cells; Figure S4: Antigen specific cytotoxicity of CAR276 expressing NK-92 cells against LN-229 target cells; Figure S5: Uptake of LNPs formulated with DiD.
