# Supplementary material for: Lipid Nanoparticle Delivery of CAR276 mRNA Enables Antigen-Specific Cytotoxicity in T Cells Against Glioblastoma Cells
Source: Biomedicines. 2026 Oct 16;14(10):2365. doi: 10.3390/biomedicines14102365 (PMC13702138; doi:10.3390/biomedicines14102365)
Supplement: Supplementary file 1 [file biomedicines-14-02365-s001.zip › biomedicines-4595721-supplementary.pdf]

biomedicines

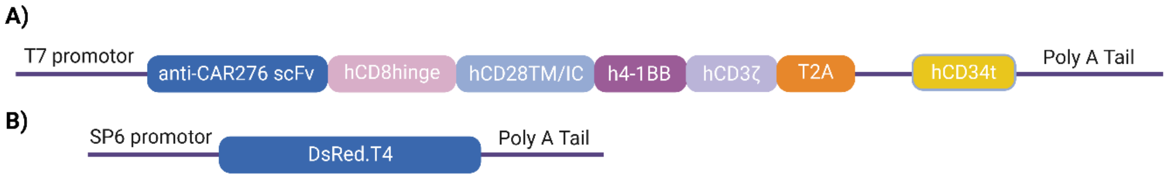


**Supplementary Figure S1.** Schematic representation of the CD276-CAR and DsRed mRNA constructs. (A) CD276-CAR mRNA construct. (B) DsRed mRNA construct.

 https://doi.org/10.3390/biomedicines14102365

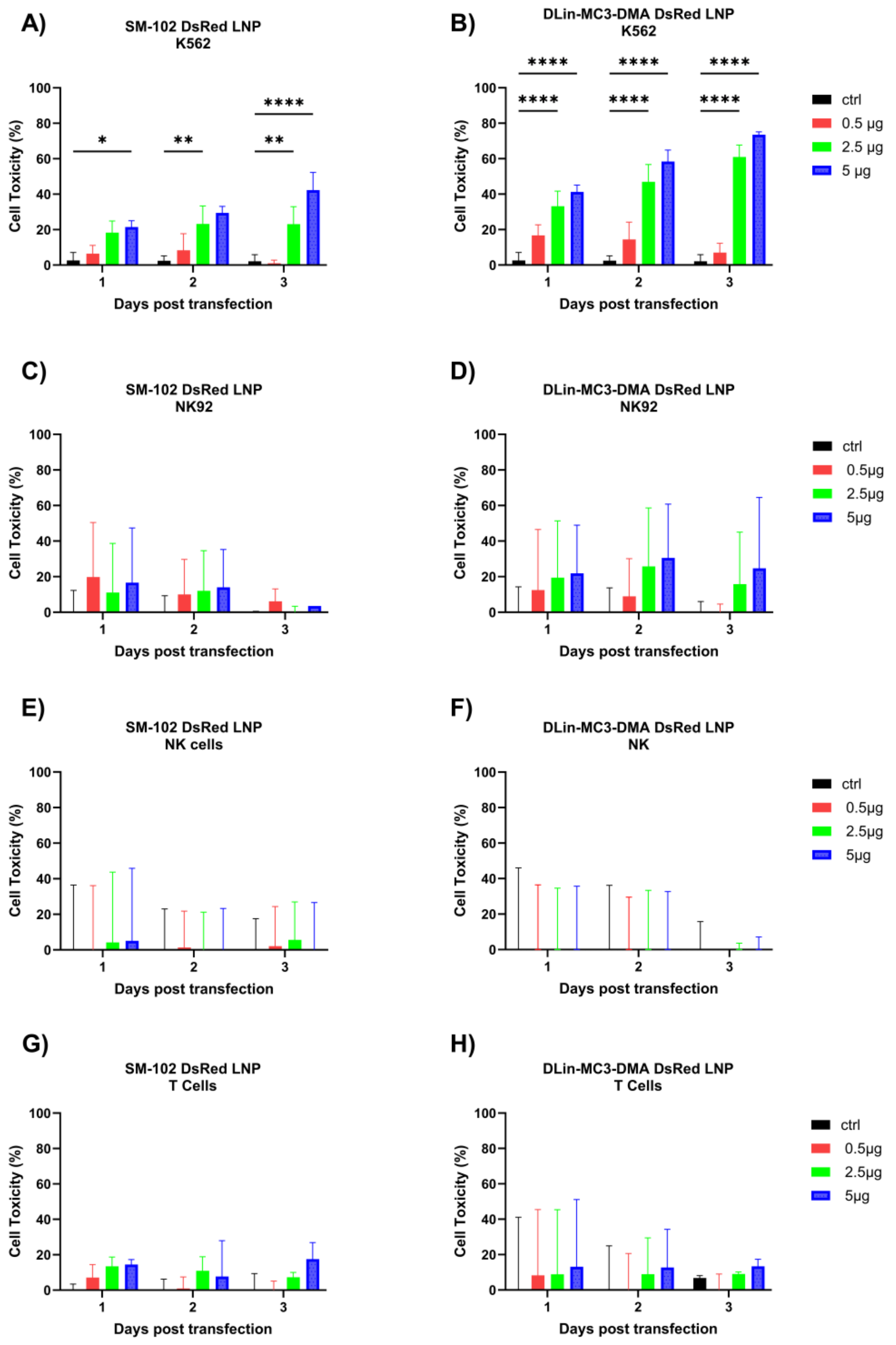


**Supplementary Figure S2.** Toxicity of LNP formulations in different cell types. Cells were transfected with different LNP formulations, and viability was assessed by flow cytometry based on the percentage of live cells. Toxicity was calculated as 100−live cells. Panels show: A & B) K562 cells (n = 3, independent experiments), C & D) NK92 cells (n = 3, independent experiments), E & F) expanded primary NK cells (n = 3, independent donors), and G & H) primary T cells (n = 3, independent donors). Statistical significance was assessed using a two-way repeated-measures ANOVA followed by Dunnett's multiple-comparisons test to compare each LNP concentration with the control at each time point. Statistical significance is indicated as follows: $P < 0.05$ (*), $P < 0.01$ (**), and $P < 0.0001$ (****).

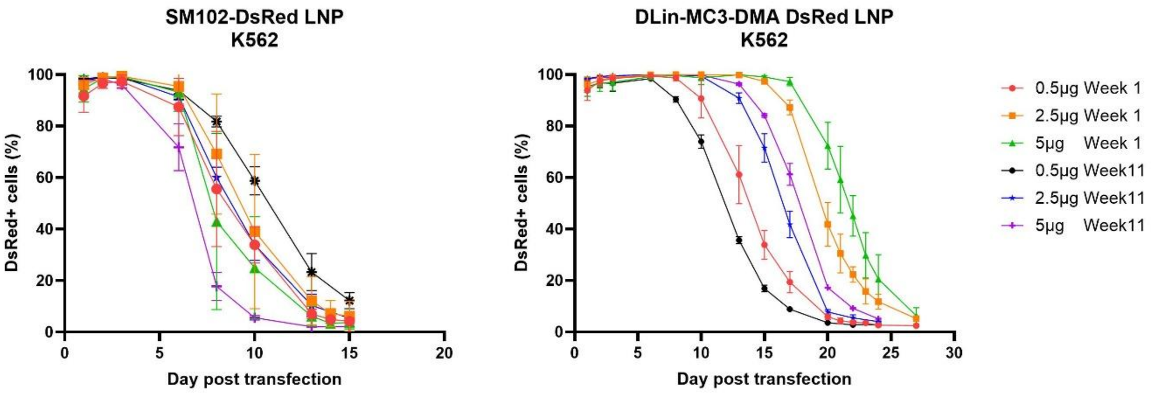


**Supplementary Figure S3.** Effect of storage on DsRed mRNA–LNP expression in K562 cells. K562 cells were transfected with lipid nanoparticles encapsulating DsRed mRNA that had been stored for either 1 week or 11 weeks after production at -20 °C . Following transfection, the expression of DsRed was evaluated to assess the impact of storage duration on LNP mediated mRNA delivery efficiency. DsRed fluorescence was quantified by flow cytometry, and the percentage of DsRed-positive cells was used as a measure of transfection efficiency. The results represent the mean ± standard deviation (SD) from three independent experiments.

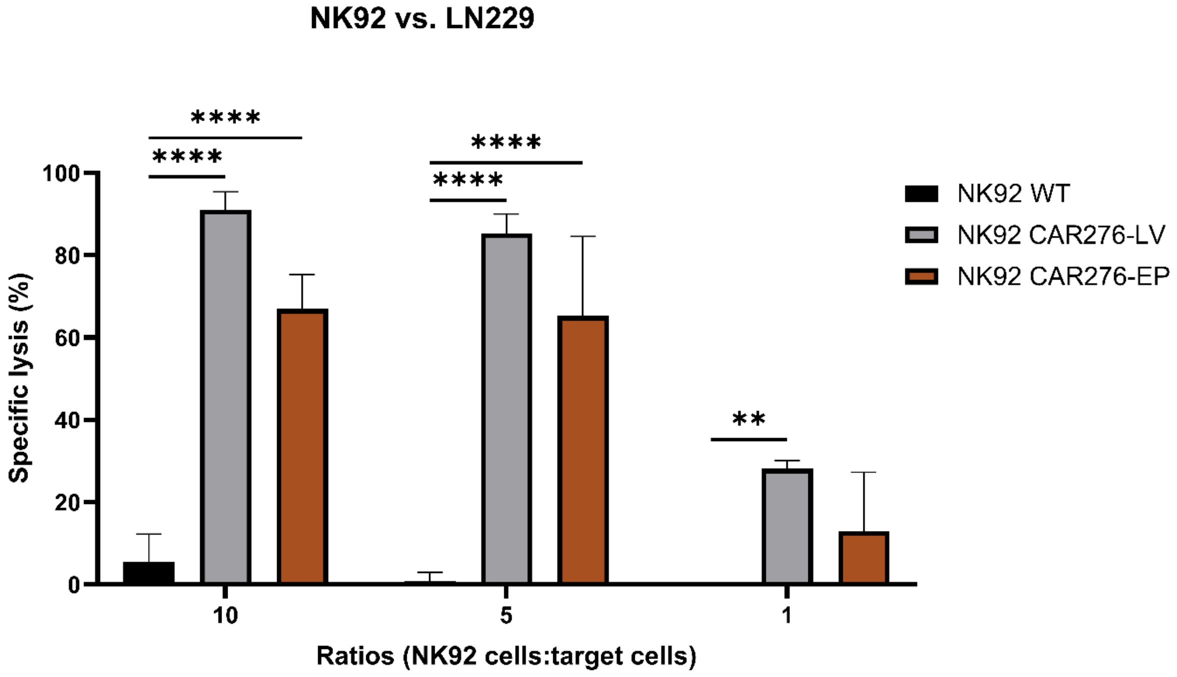


**Supplementary Figure S4.** Antigen specific cytotoxicity of CAR276 expressing NK-92 cells against LN-229 target cells. Cytotoxic activity of NK-92 cells expressing CAR276 generated either by lentiviral transduction or by mRNA electroporation was evaluated against LN-229 target cells. Effector and target cells were co-cultured with three ratios, and target cell killing was quantified

using lucifrase based killing assay after 4 hours. Data represent the mean ± SD from two independent experiments, each performed in triplicate. Statistical significance was assessed using a two-way repeated-measures ANOVA followed by Dunnett's multiple-comparisons test Statistical significance is indicated as follows: $P < 0.01$ (**), and $P < 0.0001$ (****).

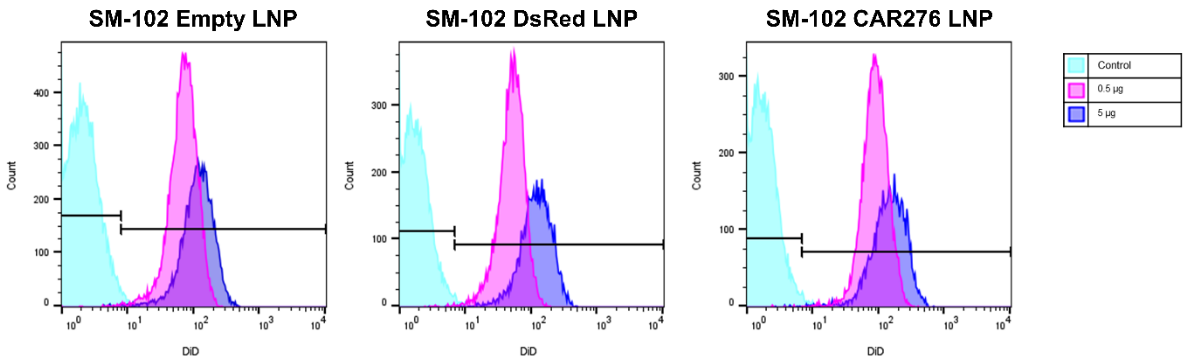


**Supplementary Figure S5.** Uptake of LNPs formulated with DiD. LNPs were stained with the lipophilic fluorescent dye DiD (MCE, USA) according to the manufacturer's instructions and incubated with *ex vivo* extended NK cells to evaluate nanoparticle uptake. Internalization was assessed by measuring DiD fluorescence using flowcytometry.

https://doi.org/10.3390/biomedicines14102365
